# Epicardial adipose tissue and severe Coronavirus Disease 19

**DOI:** 10.1186/s12933-021-01329-z

**Published:** 2021-07-20

**Authors:** Hélène Bihan, Richard Heidar, Aude Beloeuvre, Lucie Allard, Elise Ouedraogo, Sopio Tatulashvili, Yacine Tandjaoui, Stephane Gaudry, Pierre-Yves Brillet, Emmanuel Cosson

**Affiliations:** 1 Department of Endocrinology-Diabetology-Nutrition, CRNH-IdF, CINFO, AP-HP, Avicenne Hospital, Paris 13 University, Sorbonne Paris Cité, Hôpital Avicenne, 125 route de Stalingrad, 93009 Bobigny, France; 2 Unit of Radiology, Avicenne Hospital, Bobigny, France; 3 Unit of Intensive Care Medicine, Avicenne Hospital, Bobigny, France; 4 Department of Infectious Disease, AP-HP Avicenne Hospital, Bobigny, France; 5 Equipe de Recherche en Epidémiologie Nutritionnelle (EREN), Université Paris 13, Inserm (U1153), Inra (U1125), Centre d’Epidémiologie et Statistiques Paris Cité, Cnam, COMUE Sorbonne-Paris-Cité, 93017 Bobigny, France

**Keywords:** Epicardial fat volume, Epicardial adipose tissue (EAT), Inflammation, COVID-19

## Abstract

**Background:**

Both visceral adipose tissue and epicardial adipose tissue (EAT) have pro-inflammatory properties. The former is associated with Coronavirus Disease 19 (COVID-19) severity. We aimed to investigate whether an association also exists for EAT.

**Material and methods:**

We retrospectively measured EAT volume using computed tomography (CT) scans (semi-automatic software) of inpatients with COVID-19 and analyzed the correlation between EAT volume and anthropometric characteristics and comorbidities. We then analyzed the clinicobiological and radiological parameters associated with severe COVID-19 (O2 $\ge$ 6 l/min), intensive care unit (ICU) admission or death, and 25% or more CT lung involvement, which are three key indicators of COVID-19 severity.

**Results:**

We included 100 consecutive patients; 63% were men, mean age was 61.8 ± 16.2 years, 47% were obese, 54% had hypertension, 42% diabetes, and 17.2% a cardiovascular event history. Severe COVID-19 (n = 35, 35%) was associated with EAT volume (132 ± 62 vs 104 ± 40 cm^3^, p = 0.02), age, ferritinemia, and 25% or more CT lung involvement. ICU admission or death (n = 14, 14%) was associated with EAT volume (153 ± 67 vs 108 ± 45 cm^3^, p = 0.015), hypertension and 25% or more CT lung involvement. The association between EAT volume and severe COVID-19 remained after adjustment for sex, BMI, ferritinemia and lung involvement, but not after adjustment for age. Instead, the association between EAT volume and ICU admission or death remained after adjustment for all five of these parameters.

**Conclusions:**

Our results suggest that measuring EAT volume on chest CT scans at hospital admission in patients diagnosed with COVID-19 might help to assess the risk of disease aggravation.

## Background

Early in the current severe acute respiratory syndrome coronavirus 2 (SARS-CoV-2) pandemic, obesity was presumed to be one of the main risk factors for severe forms of Coronavirus Disease 19 (COVID-19) and more recent observational studies showed that between 28.6 and 47.6% of COVID-19 patients hospitalized in intensive care units (ICU) were obese [1–3]. The hypothesis for this association is that obesity, and especially visceral obesity, exacerbates COVID-19-related inflammation in patients who already have high levels of inflammation [4].

Besides obesity, impaired metabolic health (hypertension, dyslipidemia, prediabetes and insulin resistance) seems to be one of the main drivers of severe forms of COVID-19 [5]. Outside COVID-19 disease episodes, visceral obesity (defined as an excessive amount of visceral adipose tissue (VAT)) is a better marker of cardiovascular mortality and obesity-related complications than obesity measured using the body mass index (BMI) [6, 7]. In a cohort of 150 patients, Watanabe et al*.* reported a strong association between VAT area and the severity of COVID-19 [8]. However, the measurement of VAT volume requires a thoracoabdominal computed tomography (CT) scan which is usually not performed during the management of COVID-19.

Epicardial adipose tissue (EAT) volume can be measured from thoracic CT scans. EAT is located between the myocardium and the visceral pericardium and is considered the heart’s VAT [9–11]. It is a highly enriched inflammatory depot with dense macrophage, T-lymphocyte, and mass cell infiltration [12–14]. The main cytokines expressed by EAT are Tumor Necrosis Factor-alpha (TNF-alpha), interleukins 1 and 6 (IL-1, IL-6), leptin, monocyte chemoatractant protein-1 [14]. There is a correlation between EAT volume and upregulation of inflammatory markers, well demonstrated in patients with coronary artery disease [13, 14]. Moreover, this tissue expresses components of the renin angiotensin aldosterone system [12]. Just as for VAT, this fat depot have been associated with diabetes and various cardiovascular events in longitudinal studies, suggesting that it could be a link coupling diabetes, obesity and cardiovascular disease [11].

Iacobellis et al. suggested an association between EAT and COVID-19 myocarditis [15]. Furthermore, two studies [16, 17] suggested that EAT volume could be associated with severe forms of COVID-19. They included very specific populations: patients with very severe forms [16], and in the second, a cohort of Chinese patients under 40 years of age with a low proportion of obesity [17]. However, no such possible association was suggested in a third related study [30, 31]. No data on weight were available in the latter. The techniques used to assess EAT volume differed between all three studies. Given this background context, validating whether an association between EAT and severe forms of COVID-19 exists appeared essential.

Accordingly, we aimed to evaluate whether EAT volume could predict COVID-19 severity and admission to ICU or death in multiethnic patients hospitalized after being diagnosed with COVID-19. We also hypothesized that the higher the EAT volume at hospitalization, the higher the percentage of lung involvement on CT scans also at hospitalization.

## Patients and methods

### Design and data collection

This was a retrospective study performed in Avicenne University Hospital, which is located near Paris, from 7 April to 20 May 2020. Patients were consecutively enrolled in various medical departments (excluding direct ICU admission) to assess the relationship between malnutrition/metabolic profile and aggravation of COVID-19 [18].

Inclusion criteria were as follows: (i) all adult inpatients testing positive for SARS-Cov-2 infection using real-time reverse transcriptase-polymerase chain reaction in respiratory fluids, and (ii) a thoracic CT scan performed during their hospital stay. We excluded patients with severe comorbidities to avoid any potential bias due to both a poorer global prognosis, and to the risk of ICU admission following disease aggravation during hospitalization. We also excluded HIV-positive patients, as increased EAT volume has been reported in this population [19].

All patients were informed at hospital admission that their medical records could be used for research, unless they opposed. The study was approved by the medical ethics committee of Avicenne University Hospital (CLEA-2020-125). Data were analyzed anonymously.

### Study outcomes

The principal aim of our study was to evaluate whether EAT volume was associated with more severe forms of COVID-19. The primary outcome was severe COVID-19, defined as the need for high-flow nasal oxygen (at least six liters per minute) requiring a face mask [18]$.$ The secondary outcomes were: (a) a composite outcome of transfer to an ICU from a department, and (b) 25% or more $\mathrm{C}\mathrm{T}$ lung involvement. Outcomes were considered during the global hospital stay (up to 28 days after admission).

### Data collection

Data were extracted from electronic medical records and included anthropometric characteristics (age, sex, ethnicity, waist circumference, BMI) and comorbidities.

Ethnicity was reported as Caucasian, Arabic (Middle East, Maghreb), Afro-Caribbean (African, African American, Caribbean), Asian (Asian continent). Weight status was defined as: underweight (less than 18.5 kg/m^2^), normal weight (18.5–24.9), overweight ($\ge$ 25), obese ($\ge$ 30), with a specific obesity threshold for Asian individuals (< 18.5; 18.5–23, $\ge$ 23 and $\ge$ 27.5 kg/m^2^, respectively) [20]. Apart from obesity, four other comorbidities, specifically hypertension, diabetes, cardiovascular disease (i.e., myocardial infarction, stroke), and chronic lung disease, were self-reported or inferred from the use of, respectively, blood pressure, blood glucose, lipid lowering agents, and treatment for chronic lung disease. Data were routinely collected for the following biomarkers on plasma from fasting individuals: white cells (*10^3^/µl), C-reactive protein (CRP) (mg/dl) using immunoturbidimetry with anti-CRP monoclonal antibodies on latex particles, procalcitonin (PCT) (µg/l) using fluorometric sandwich immunoassay, ferritinemia (ng/ml) using immunoturbidimetry on latex particles, d-Dimers (ng/ml) using a sandwich immunoassay, fibrinogen (chronometry-Thrombin Dade) and finally, albuminemia (g/l). A Cobas 6000 analyzer (Roche diagnostics) was used for all biomarkers except fibrinogen where a Siemens analyzer was used.

Albumin can result from both inflammation and malnutrition and was therefore adjusted for CRP using the following formula: albumin (g/l) = measured albumin (g/l) + (CRP (mg/l) − 15)/25. This formula is based on the fact that when CRP is above 15 mg/l, albumin decreases by 1 g/l per 25 mg/l increase in CRP [21].

#### Lung involvement and EAT volume measurement

GE (Healthcare Digital, France) or Siemens (Healthineers, France) scanners were used for all CT scans. The extent of typical COVID-19 lesions on chest CT scans was categorized into five groups: 0: no involvement, 1: minimal involvement (i.e., < n 25%), 2: $\ge$ 25–50%, 3: 51–75%, 4: > 75%. Total adipose tissue around the heart (i.e., pericardial adipose tissue) comprises EAT and paracardial adipose tissue. They are separated by the pericardium, which is not easy to identify [22]. EAT volume was measured using a semi-automatic segmentation technique with a new software package ‘AW VolumeShare 7’ which is currently being developed by GE (Healthcare Digital, France). The quantity of EAT depot contained in the epicardium and surrounding the coronary artery trees was assessed examining all the axial slices from the thoracic inlet to the beginning of the abdomen. The software automatically delimited the EAT volume (in cm^3^) by summing appropriate pixels using a CT Hounsfield unit (HU) range of between − 150 to − 50 HU. The software user could readjust the delimitation manually when necessary. These data were collected by IR (see acknowledgments).

### Statistical analysis

Baseline continuous variables were expressed as mean ± standard deviation (SD). Normality was assessed using the Skewness-Kurtosis test. Categorical variables were expressed as frequencies (percentages). Continuous variables were compared using the Kruskal–Wallis test and categorical variables with the chi-squared (χ^2^) or Fisher’s exact test (depending on the number of patients within each group). All tests were two-sided and had a p-value significance level of 0.05. To assess correlations between two quantitative variables, the non-parametric Spearman coefficient was used.

Univariable and multivariable logistic regression models were performed to analyze the relationship between EAT volume (per 10 cm^3^) and the three study outcomes with the following models: model 1: unadjusted; model 2: adjusted for age; model 3: adjusted for sex; model 4: adjusted for BMI; model 5: adjusted for waist circumference; model 6: adjusted for ferritinemia; model 7: adjusted for CT lung involvement ≥ 25%. Models 8 and 9 included age, sex and BMI (forced variables) and covariates that were associated with the primary outcome (i.e., severe COVID-19) in univariate analysis (p < 0.05). For model 8, these were ferritinemia, and 25% or more CT lung involvement, while for model 9, they were hypertension and 25% or more CT lung involvement. We also tested for interactions between EAT volume and both age and sex, separately. Analyses were conducted using STATA software (version 14.2, Texas, USA).

## Results

### Population characteristics

Of the 109 consecutive inpatients who had a CT scan upon hospitalization, we excluded 7 patients (3 with severe comorbidities, 4 HIV-positive patients). EAT volume was measured in 100 of the remaining 102 patients. Table 1 shows the characteristics of our study population. Mean age was 61.7 ± 16.2 years. The percentages of obesity, hypertension, diabetes were higher than 40%.

**Table 1:** Characteristics of the study population according to oxygen flow requirement

	Available data	Total population	O_2_ < 6 l/min	O_2_ $\ge$ 6 l/min	p
	n	100	65	35	
Age (years)	100	61.7 ± 16.2	58.7 ± 15.8	67.5 ± 15.5	**0.004**
Sex (male)	100	63 (63.0)	37 (56.9)	26 (74.3)	0.09
Ethnicity	100				
Caucasian		32 (32)	17 (26)	32 (32)	
Arabic		14 (14)	12 (18)	14 (6)	
Afro-Caribbean		34 (34)	23 (35)	11 (31)	
Asian		14 (14)	11 (17)	3 (9)	
Other		6 (6)	2 (3)	4 (11)	0.08
Body mass index (kg/m^2^)	99	28.9 ± 6.26	29.2 ± 6.1	28.5 ± 6.6	0.85
Waist circumference (cm)	91	105.3 ± 16.2	103.5 ± 17.7	109.8 ± 10.9	0.01
Comorbidities					
Obesity	99	47 (47)	31 (47.7)	16 (45.7)	0.39
Hypertension	100	54 (54)	31 (47.7)	23 (65.7)	0.09
Diabetes	100	42 (42)	24 (36.9)	18 (51.4)	0.16
Cardiovascular disease	99	17 (17.2)	9 (14.1)	8 (22.9)	0.28
Lung disease	96	5 (5.2)	3 (4.7)	2 (6.1)	0.79
Biological parameters					
White cells (*10^3^/µl)	100	7.1 ± 2.8	7.0 ± 2.9	7.2 ± 2.6	0.55
C-reactive protein (mg/dl)	100	82.3 ± 85.4	72.8 ± 83.4	100.0 ± 87.5	0.06
Procalcitonin (µg/l)	97	0.7 ± 2.3	0.47 ± 1.7	0.99 ± 3.0	0.07
Ferritinemia (ng/ml)	86	1128 ± 2157	723 ± 735	1812 ± 3326	**0.003**
d-Dimers (ng/ml)	97	1629 ± 2352	1353 ± 1366	2119 ± 3447	0.06
Albuminemia (g/l)	89	33.4 ± 5.0	34.2 ± 4.7	32.0 ± 5.2	0.06
Computed tomography parameters					
≥ 25% lung involvement	99	18 (18.2)	7 (10.8)	11 (32.4)	**0.01**
≥ 50% lung involvement	99	6 (6.1)	2 (3.1)	4 (11.8)	**0.03**
EAT volume (cm^3^)	100	114 ± 50	104 ± 40	132 ± 62	**0.02**

Data are n (%) or mean ± standard deviation or n (%); p-values < 0.05 are identified in bold case

*EAT* epicardial adipose tissue

Severe COVID-19 was diagnosed in 35 patients after a mean time of 7 ± 4 days between the first symptoms and hospitalization. Following disease aggravation, 11 patients were transferred from non-ICU departments to the ICU (11%) where one subsequently died. Three other patients died in non-ICU departments. The four deceased patients were aged between 83 to 89 years old. A total of 18 patients had 25% or more CT scan lung involvement upon hospitalization.

### Determinants of EAT volume

EAT volume was higher in men than in women, and in individuals with a history of hypertension than in those with no such history (Table 2). It was positively correlated with age, waist circumference and ferritin level, but negatively correlated with albumin level (Table 2).

**Table 2:** Determinants of epicardial adipose tissue (EAT) volume

Parameters	Modalities	n	EAT volume	p*
Sex	Male	63	130 ± 48	**0.0001**
	Female	37	87 ± 43	
Ethnicity	Caucasian	32	133 ± 60	0.09
	Arabic	14	104 ± 39	
	Afro-Caribbean	34	110 ± 41	
	Asian	14	93 ± 40	
	Other	6	104 ± 71	
Obesity	No	52	113 ± 49	0.5
	Yes	47	115 ± 53	
Hypertension	No	46	102 ± 46	**0.007**
	Yes	54	124 ± 52	
Diabetes	No	58	109 ± 51	0.10
	Yes	42	121 ± 49	
Cardiovascular disease	No	82	112 ± 50	0.23
	Yes	17	124 ± 52	
Continuous variables			Coefficient	p
Age (years)			0.4	**0.000**
Body mass index (kg/m^2^)			0.09	0.32
Waist circumference (cm)			0.30	**0.004**
White Cells (*103/µl)			0.08	0.42
C-reactive protein (mg/dl)			− 0.03	0.74
Ferritinemia (ng/ml)			0.28	**0.01**
d-Dimers (ng/ml)			0.04	0.71
Albuminemia (g/l)			− 0.25	**0.02**

p-values < 0.05 are identified in bold case

* Spearman’ s correlation

*EAT* epicardial adipose tissue

### Parameters associated with severe COVID-19

Patients with severe COVID-19 were older, had a higher ferritin level, a higher EAT volume, and more severe lung involvement than those without severe COVID-19 (Table 1).

The association between EAT volume (per 10 cm^3^: odds ratio 1.12 (95% confidence interval 1.03–1.23), p = 0.01) and severe COVID-19 remained significant after separate adjustment for sex, BMI, waist circumference, ferritinemia and CT lung involvement. However, it disappeared after adjustment for age (p = 0.07) separately, and after adjustment for age, sex, BMI, ferritinemia and lung involvement combined (p = 0.16) (Table 3).

**Table 3:** Risk of adverse outcomes per 10 cm^3^ increase of EAT volume

Models	O_2_ $\ge$ 6 l/min	Admission to ICU or death
1. Unadjusted	1.12 (1.03–1.23), p = **0.01**	1.18 (1.05–1.32), p = **0.005**
2. Adjusted for age	1.09 (0.99–1.20), p = 0.07	1.17 (1.03–1.32), p = **0.014**
3. Adjusted for sex	1.11 (1.01–1.22), p = **0.03**	1.15 (1.03–1.30), p = **0.018**
4. Adjusted for BMI	1.13 (1.03–1.24), p = **0.01**	1.19 (1.06–1.34), p = **0.004**
5. Adjusted for waist circumference	1.15 (1.03–1.28), p = **0.01**	1.12 (0.98–1.28), p = 0.09
6. Adjusted for ferritinemia	1.14 (1.03–1.26), p = **0.01**	1.19 (1.06–1.35), p = **0.005**
7. Adjusted for CT lung involvement ≥ 25%	1.15 (1.04–1.26), p = **0.004**	1.19 (1.06–1.34), p = **0.004**
8. Adjusted for age, sex, BMI, ferritinemia and lung involvement ≥ 25%	1.11 (0.96–1.27), p = 0.16	–
9. Adjusted for age, sex, BMI, hypertension and lung involvement ≥ 25%	–	1.20 (1.01–1.41), p = **0.03**

p-values < 0.05 are identified in bold case

### Parameters associated with transfer to ICU and/or mortality

The 14 patients who were transferred to ICU and/or who died were more likely to have hypertension, 25% or more CT lung involvement, and a higher EAT volume (153 ± 45 vs 108 ± 67 cm^3^, p = 0.015) than the other patients (Table 4). EAT volume was associated with the ICU/mortality composite variable (odds ratio per 10 cm^3^ 1.18 (1.05–1.32), p = 0.005). Multivariable analyses demonstrated that this association was independent of age, sex, BMI, ferritinemia, CT lung involvement when taken separately, and for age, sex, BMI, hypertension and lung involvement when combined (Table 3).

**Table 4:** Characteristics of the study population according to the composite admission to ICU/mortality outcome

	No admission to ICU or death	Admission to ICU or death	p
	n = 86	n = 14	
Age (years)	60.9 ± 16.1	67.2 ± 16.1	0.23
Sex (male)	51 (59.3)	12 (85.7)	0.06
Ethnicity			0.74
Caucasian	27 (31)	5 (36)	
Arabic	13 (15)	1 (7)	
Afro-Caribbean	28 (33)	6 (43)	
Asian	12 (14)	2 (14)	
Other	6 (7)	0 (0)	
Body mass index (kg/m^2^)	29.0 ± 6.4	28.4 ± 5.4	0.68
Waist circumference (cm)	105.0 ± 16.8	107.5 ± 11.6	0.41
Comorbidities			
Obesity	43(50)	4(28.6)	0.28
Hypertension	43(50)	11(78.6)	**0.047**
Diabetes	36(41.9)	6(42.9)	0.94
Cardiovascular disease	15(17.7)	2(14.3)	0.76
Lung disease	3(3.7)	2(14.3)	0.10
Biological parameters			
White cells (*10^3^/µl)	7.0 ± 2.8	7.8 ± 2.7	0.22
C reactive protein (mg/dl)	77.5 ± 82.5	111.7 ± 100.3	0.24
Procalcitonin (µg/l)	0.4 ± 1.5	2.0 ± 4.6	0.13
Ferritinemia (ng/ml)	883 ± 854	2386 ± 4938	0.06
d-Dimers (ng/ml)	1541 ± 2353	2150 ± 2364	0.13
Albuminemia (g/l)	33.5 ± 5.0	32.6 ± 5.2	0.58
Radiological parameters			
≥ 25% lung involvement	10 (11.8)	8 (57.1)	**0.000**
EAT volume (cm^3^)	108 ± 47	153 ± 67	**0.015**

p-values < 0.05 are identified in bold case

### Parameters associated with lung involvement

The 18 patients who had 25% or more $\mathrm{C}\mathrm{T}$ lung involvement had similar parameter values to patients with less lung involvement, apart from a higher CRP level (Table 5). Interestingly, EAT volume was not associated with 25% or more CT lung involvement (115 ± 48 vs 116 ± 60 cm^3^, p = 0.95).

**Table 5:** Characteristics of the study population according to computed tomography lung involvement

	< 25% CT lung involvement	$\ge$ 25% CT lung involvement	p
	n = 81	n = 18	
Age (years)	61.2 ± 15.4	62.9 ± 19.2	0.81
Sex (male)	50 (61.7)	13 (72.2)	0.40
Ethnicity			
Caucasian	28 (35)	4 (22)	
Arabic	13 (16)	1 (6)	
Afro-Caribbean	26 (32)	8 (44)	
Asian	9 (11)	5 (28)	
Other	5 (6)	0	0.17
Body mass index (kg/m^2^)	29.2 ± 6.1	28.7 ± 6.6	0.75
Waist circumference (cm)	105.3 ± 16.6	105.6 ± 14.4	0.73
Comorbidities			
Obesity	38 (47)	9 (50)	0.88
Hypertension	40 (49.4)	13 (72.2)	0.08
Diabetes	36 (44.4)	5 (27.8)	0.19
Cardiovascular disease	6 (7.4)	1 (5.6)	0.78
Lung disease	4 (5.1)	1 (5.6)	0.94
Biological parameters			
White cells (*10^3^/µl)	6.9 ± 2.7	8.1 ± 3.0	0.06
C-reactive protein (mg/dl)	72.7 ± 83.2	125.8 ± 86.4	**0.01**
Procalcitonin (µg/l)	0.4 ± 1.6	1.6 ± 4.1	0.10
Ferritinemia (ng/ml)	1101 ± 2391	1268 ± 1004	0.09
d-Dimers (ng/ml)	1550 ± 2400	1970 ± 2233	0.40
Albuminemia (g/l)	33.5 ± 5.1	33.1 ± 5.1	0.95
Radiological parameters			
EAT volume (cm^3^)	115 ± 48	116 ± 60	0.95

Data are n (%) or mean ± standard deviation; p-values < 0.05 are identified in bold case

*CT* computed tomography, *EAT* epicardial adipose tissue

## Discussion

The association we found between EAT and severe COVID-19 was dependent on age, but not dependent on inflammatory or radiological parameters. Admission to ICU or death was associated with a higher EAT volume, independently of all confounders.

### Correlation between EAT volume and risk factors

In line with previous findings, EAT volume was significantly associated with age [23] and with the presence of some cardiovascular risk factors, such as hypertension [11, 22]. Unlike the literature, it was not higher in people with cardiovascular personal history [11] or obesity [24]. However, it was associated with waist circumference, reflecting findings elsewhere [25]. BMI in our population was higher than in most studies that have evaluated EAT volume [24, 26]. Park et al. demonstrated that the association between EAT thickness and metabolic syndrome was stronger in Asian patients with a BMI < 27 kg/m^2^ [27]. An autopsy-based study found that the association between EAT volume and waist circumference was much stronger than its association with BMI [23]. Finally, ethnicity influences the association between EAT and BMI [28].

### Determinants of severe forms of COVID-19

As in previous studies, we found that age, inflammatory markers (specifically, ferritinemia and CRP), and lung involvement were associated with severe COVID-19 in our study sample [2, 3]. However, unlike many other studies, neither male gender nor obesity was associated with severe outcomes [1, 3]. Apart the relatively small size of our cohort, this result might be explained by the fact that we selected inpatients who were not hospitalized directly into the ICU department [29]. Only 6% of them had CT lung involvement > 50%.

### EAT volume and severe forms of COVID-19

The principal findings of our study are the associations between EAT volume and severe COVID-19, and between EAT volume and transfer from a non-ICU medical department to the ICU or death.

Results from three other recent studies have suggested an association between EAT amount and severe forms of COVID-19 in very different populations (younger, high-risk, and monoethnic) and using different definitions for severe forms of COVID-19 [14–16]. We describe these studies below.

First, Deng et al. showed an association between EAT volume and the need for oxygen in young Chinese patients (18–40 years; n = 65), with low percentages of risk factors and obesity [17]. All patients with an EAT volume lower that 105 cm^3^ had moderately severe COVID-19. In our study, few patients were younger than 40 years and the relationship between EAT and severe COVID-19 disappeared after adjustment for age.

Second, Grodecki et al*.* also reported an association between EAT volume and CT lung involvement, admission to ICU, invasive mechanical ventilation, vasopressor therapy, and death, in an international registry [16]. These patients, selected because scans highlighted diffuse lung lesions, had severe comorbidities [chronic lung disease (10–26%), heart failure (13–39%), coronary artery disease (23–43%)]; these patients had high intubation (43%) and mortality (56%) rates [16]. The EAT volumes were 132 cm^3^ (88–158 cm^3^) in the patient group with the poorest prognosis, and 85 cm^3^ (58–129 cm^3^) in the group with the best prognosis [16].

Third, Abrishami et al*.* found discordant results on the influence of EAT on COVID-19 prognosis in Iranian patients [30, 31]. EAT volume was only associated with upper-zone lung involvement, but not with death. Lower EAT density was a marker of poor disease prognosis/death [30]. This association disappeared in their multivariable model. Furthermore, the authors had no data on BMI.

Given the heterogeneity between all the cohorts in the above studies and our own, further research is needed to investigate whether certain specific factors modify the association between EAT and prognosis. From our findings and those of the above-mentioned studies, age, ethnicity, obesity and lung involvement seem to be good candidates for such research.

### Explanatory hypotheses

How can we explain the association we found between EAT volume and severe forms of COVID-19? COVID-19 infection, diabetes, and obesity seem to share some common metabolic and inflammation reaction pathways [27, 29, 32–34]. EAT, which is a white adipose tissue, has the highest rates of lipogenesis and fatty acid metabolism among all visceral fat depots, and displays metabolic, thermogenic, and mechanical properties [11]. In addition to studies on cytokines expression in EAT [13, 14], others studies have shown an association between EAT volume and the plasmatic level of certain cytokines (IL-15, IL-15Rα [35] and IL-13 [36]), but no correlation with others [37]. Obese patients display higher circulating level of cytokines [38], as higher expression of cytokines in their EAT [39]. Accordingly, we can hypothesize that in patients already at risk, the higher the production of cytokines by EAT, the more intense the cytokine storm.

Higher EAT volumes have been associated with cardiovascular disease and atherosclerosis in patients with diabetes and chronic kidney disease [11, 40, 41]. In COVID-19 infection, 7 and 35% of patients with COVID-19 suffer from cardiac injury [42]; and some authors have suggested that EAT may be a major contributor to SARS-CoV-2 entering the heart [15, 39, 43], and could be a good predictor of adverse events in patients with a history of cardiovascular disease.

The association between EAT and ferritinemia which we found has not been previously published. This association begs the question of causality. Could it be the case that the EAT amount influences both immune response and production of ferritin by macrophages, in response to infection?

The inverse relationship between albumin and EAT suggests that nutritional status and EAT may be related. Studies have reported reduced EAT thickness 12 months after bariatric surgery [44, 45]. Accordingly, we need data on weight evolution during a COVID-19 infection to understand the inverse correlation.

### Strengths and limitations

This study was monocentric, assuring that the treatment received was similar for all COVID-19 patients during the period of time considered. Furthermore, the fact that we measured BMI and waist circumference for all patients and included them in the multivariable analyses enabled us to evaluate the influence of EAT on COVID-19 severity prognosis more clearly. The database we used included multiple inflammatory variables, including albumin adjusted for CRP level in order to take into account malnutrition with a view to ensuring model robustness [18].

Furthermore, the technique used to measure EAT volume was robust; by using several axial slices (in cm^3^), we were able to establish the amount of fat much more precisely. EAT volume is often measured with automatic or semi-automatic software because manual methods require excessively long processing times and readings by a radiologist. CT-quantification is challenging and may have different sensitivity and specificity depending on the software used. In contrast, in our study, the pericardial line (which delimits underneath the epicardial fat) was automatically identified by the software and was manually readjusted if necessary. Only 7% of CT scans were readjusted by the reader because of minor inaccuracies.

Our study has limitations. The lack of association between EAT volume and BMI may suggest selection bias in the population hospitalized for COVID-19. It may be that rapid weight loss could modify the relationship between BMI, EAT and disease prognosis. As a standardized method for EAT quantification has not yet been determined, different studies have used different Hounsfield unit thresholds to delimit EAT volume [46]. Accordingly, there are no clear threshold values for the physiological or pathological levels of EAT. Moreover, we did not measure EAT attenuation, something which has been suggested elsewhere as a more precise measure of EAT implication in inflammation [47].

Finally, our cohort was relatively small. Nevertheless, multivariable adjustments were possible.

## Conclusion

In this cohort with a high rate of obese patients, mean EAT volume was associated with severity of COVID-19 and with transfer from a non-ICU hospital medical department to ICU or death. However, we found no association with lung involvement. Leaving the question of causality aside, it is possible that inflammatory properties of EAT increase systemic inflammation. Further research needs to investigate whether or not the impact of EAT volume on COVID-19 prognosis is dependent on age, sex, BMI or cardiovascular history. Measuring EAT volume upon hospitalization could be useful to precisely determine the prognosis of COVID-19.

## Data Availability

The datasets used and/or analyzed during the current study are available from the corresponding author on reasonable request.

## Abbreviations

BMI: Body mass index
CLEA: Local Committee of Ethics
CT: Computed tomography
COVID-19: Coronavirus Disease 19
CRP: C-reactive protein
EAT: Epicardial adipose tissue
HIV: Human immunodeficiency virus
HU: Hounsfield unit
ICU: Intensive care unit
SD: Standard deviation
SARS-Cov2: Severe acute respiratory syndrome coronavirus 2
TNF-alpha: Tumor necrosis factor-alpha
VAT: Visceral adipose tissue
